# Detection method of wheat spike improved YOLOv5s based on the attention mechanism

**DOI:** 10.3389/fpls.2022.993244

**Published:** 2022-09-28

**Authors:** Hecang Zang, Yanjing Wang, Linyuan Ru, Meng Zhou, Dandan Chen, Qing Zhao, Jie Zhang, Guoqiang Li, Guoqing Zheng

**Affiliations:** ^1^Institute of Agricultural Economics and Information, Henan Academy of Agricultural Sciences, Zhengzhou, China; ^2^Key Laboratory of Huang-Huai-Hai Smart Agricultural Technology, Ministry of Agriculture and Rural Affairs, Zhengzhou, China; ^3^College of Life Sciences, Zhengzhou Normal University, Zhengzhou, China; ^4^College of Computer and Information Engineering, Henan Normal University, Xinxiang, China

**Keywords:** wheat, spike number detection, attention mechanism, deep learning, YOLOv5s

## Abstract

In wheat breeding, spike number is a key indicator for evaluating wheat yield, and the timely and accurate acquisition of wheat spike number is of great practical significance for yield prediction. In actual production; the method of using an artificial field survey to count wheat spikes is time-consuming and labor-intensive. Therefore, this paper proposes a method based on YOLOv5s with an improved attention mechanism, which can accurately detect the number of small-scale wheat spikes and better solve the problems of occlusion and cross-overlapping of the wheat spikes. This method introduces an efficient channel attention module (ECA) in the C3 module of the backbone structure of the YOLOv5s network model; at the same time, the global attention mechanism module (GAM) is inserted between the neck structure and the head structure; the attention mechanism can be more Effectively extract feature information and suppress useless information. The result shows that the accuracy of the improved YOLOv5s model reached 71.61% in the task of wheat spike number, which was 4.95% higher than that of the standard YOLOv5s model and had higher counting accuracy. The improved YOLOv5s and YOLOv5m have similar parameters, while RMSE and MEA are reduced by 7.62 and 6.47, respectively, and the performance is better than YOLOv5l. Therefore, the improved YOLOv5s method improves its applicability in complex field environments and provides a technical reference for the automatic identification of wheat spike numbers and yield estimation. Labeled images, source code, and trained models are available at: https://github.com/228384274/improved-yolov5.

## Introduction

Wheat is an important food crop in our country. In 2021, the planting area of wheat will be 22.911 million hectares, and the output will be 134 million tons in our country; China is the largest wheat producer in the world (Sreenivasulu and Schnurbusch, 2012; Ge et al., 2018; Chen et al., 2021; Wen et al., 2022). However, the current COVID-19 epidemic is raging, the domestic and foreign environments are complex and changeable, abnormal weather and natural disasters are frequent, and food security is facing severe challenges (Laborde et al., 2020; FAO, 2021; Ministry of Emergency Management of the People’s Republic of China, 2022). The spike number is an important indicator for wheat yield estimation (Zhang et al., 2007; Gou et al., 2016; Zhou et al., 2021). Therefore, wheat spike number detection is the key to predicting and evaluating wheat yield. Timely and accurate acquisition of wheat spike numbers has always been the focus of wheat breeding and cultivation research.

In actual production, the acquisition of wheat spikes mainly includes low-throughput artificial field investigation and high-throughput remote sensing image processing. Artificial field surveys have the disadvantages of strong subjectivity, strong randomness, and lack of uniform standards, which lead to the shortcomings of time-consuming, labor-intensive, and low-efficiency researchers. They cannot obtain statistical results of wheat spikes efficiently and quickly (Kamilaris and Prenafeta-Boldú, 2018). The high-throughput remote sensing image processing is based on the feature fusion of different textures (Ganeva et al., 2022), color features (Grillo et al., 2017), spectral reflectance, and uses machine learning to detect targets in wheat spike images to extract the number of wheat spikes. Zhao et al. (2021) proposed a method based on an improved YOLOv5, which can accurately detect the number of wheat spikes in unmanned aerial vehicle (UAV) images; the average accuracy (AP) of wheat spike detection in UAV images is 94.1%, which is 10.8% higher than the standard YOLOv5, and solves the problem of the wrong detection and missed detection of wheat spikes due to occlusion conditions. Gong et al. (2021) proposed a method of wheat-head detection based on a deep neural network to enhance the speed and accuracy of detection; the mean average precision of the proposed method is 94.5%, and the detection speed is 71 FPS. Li et al. (2022) used a deep-learning algorithm (Faster R-CNN) on red green blue (RGB) images to explore the possibility of image-based detection of spike numbers and its application to identify the loci underlying spike numbers. Xiong et al. (2019) proposed a simple yet effective contextual extension of TasselNet–TasselNetv2, which simultaneously addresses two important use cases in plant counting.

Alkhudaydi et al. (2019a) developed a deep-learning-based analysis pipeline to segment spike regions from complicated backgrounds. Zhao et al. (2022) proposed a deep learning method for oriented and small wheat spike detection (OSWSDet); the AP is 90.5%. Wang Y. D. et al. (2021) proposed an improved EfficientDet-D0 object detection model for wheat ear counting; the counting accuracy of the improved EfficientDet-D0 model reaches 94%, which is about 2% higher than the original model and focuses on solving occlusion. Wang et al. (2019) proposed a field-based high-throughput phenotyping approach using deep learning that can directly measure morphological and developmental phenotypes in genetic populations from field-based imaging. David et al. (2020, 2021) built the Global Wheat Head Detection (GWHD) dataset and released in 2021 a new version of the GWHD dataset, which is bigger, more diverse, and less noisy than the GWHD_2020 version. Yang et al. (2021) proposed an improved YOLOv4 with a spatial and channel attention model was proposed that could enhance the feature extraction capabilities of the network by adding receptive field modules. Fernandez-Gallego et al. (2018) proposed an automatic algorithm for the number of wheat spikes to estimate the number of wheat spikes under field conditions. Lu et al. (2017) developed a smartphone application software to complete the detection and collection of wheat diseased spikes, with an accuracy of 96.6%. Pound et al. (2017) used the deep learning method to calculate the number of wheat spikes through the images of wheat spikes taken under greenhouse conditions. Hasan et al. (2018) and Li et al. (2021) use the R-CNN method to detect, count, and analyze wheat spikes, which has high recognition accuracy, but the detection speed is slow and cannot be deployed in real-time detection equipment. Compared with the above methods, our proposed method has a faster detection speed while improving accuracy than the two-stage target detection method. Compared with other improved YOLO algorithms, we introduce the attention mechanism into the YOLO model to improve the network’s ability to extract the target features, rather than relying on data sets. Compared with the traditional image processing methods, the deep learning technology can automatically extract the target features, while the traditional methods mainly rely on manual design features, and the algorithm has no generalization. The extraction ability of unknown features is poor. Therefore, we introduce the attention mechanism into the YOLO model to ensure accuracy and faster detection speed, which lays the foundation for future deployment on mobile devices.

In recent years, with the rapid development of artificial intelligence, deep learning algorithms have been widely used in the industrial field. Huang et al. (2021) determined whether workers meet the standard of wearing helmets by improving the YOLOv3 algorithm. The final result is that the mAP reaches 93.1%. Huang et al. (2022) used the improved single shot multiBox detector (SSD) algorithm to verify the effectiveness of multi-scale feature fusion for small targets. Sun et al. (2022) solved the problems of poor image quality, loss of detail information, and excessive brightness enhancement in the image enhancement process in a low-light environment by improving the multi-scale Retinex and ABC algorithms. Bai et al. (2022) improved the network by combining the target frame recommendation strategy in the SSD algorithm with the frame regression algorithm to improve the detection accuracy of small targets. Weng et al. (2021) proposed an angle network model to accurately estimate the robot picking angle, which improves the accuracy and real-time detection. Gao et al. (2019) applied deep neural networks to hand detection and achieved good results. The deep learning object detection model has made remarkable progress in wheat spike image detection (Madec et al., 2019; He et al., 2020), which is the main technical means for wheat spike recognition and detection counting, and has reached top performance in detection accuracy and speed (Zhou et al., 2018a; Khoroshevsky et al., 2021; Lu et al., 2021; Wang D. et al., 2021). Single-stage algorithms for object detection include SSD (Liu et al., 2016) and the YOLO family, which includes YOLO (Redmon et al., 2016), YOLO9000 (Redmon and Farhadi, 2017), YOLOv3 (Redmon and Farhadi, 2018), YOLOv4 (Bochkovskiy et al., 2020), and YOLOv5 (Ultralytics, 2021). The single-stage detection algorithm is also known as the target detection algorithm based on regression analysis, which regards the target detection problem as a regression analysis problem on target location and category information, which can directly output the detection results through a neural network model. Considering the cost and observational limitations of satellites, ground-based remote sensing, and drones according to the needs of researchers, the use of smartphones has significantly improved the efficiency of wheat spike surveys. However, in the detection of wheat spike images, due to the high density of wheat spike, serious occlusion, and serious cross-overlapping, detection errors and missed detection of the wheat spike are caused. At the same time, due to the large morphological differences between individual wheat spikes and the fact that the color of the wheat spike is consistent with the background, the difficulty and accuracy of wheat spike detection are further increased.

In order to solve the above problems, this paper proposes an improved YOLOv5s target detection method using an attention mechanism for the accurate detection of wheat spikes. This method introduces ECA into the C3 module of the backbone structure of the YOLOv5s network model; GAM is inserted between the neck structure and the head structure; the attention mechanism can more effectively extract feature information and suppress useless information. This method improves the applicability of the YOLOv5s method in complex field environments, which can accurately detect the number of small-scale wheat spikes and better solve the problem of occlusion and overlap of a wheat spike.

## Materials and methods

### Overview of the test site

**FIGURE 1:**
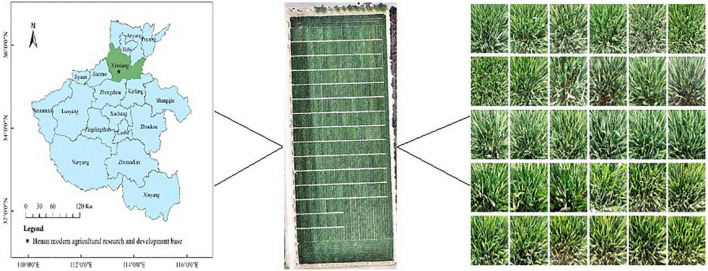
Geographical location of the study area.

The experimental site is located in the regional wheat experiment of the Henan Modern Agriculture Research and Development Base of the Henan Academy of Agricultural Sciences. It is located at 35°0′44″ north latitude and 113°41′44″ east longitude, as shown in Figure 1. The climate type is a warm temperate continental monsoon climate, with an annual average temperature of 14.4°C, annual average rainfall of 549.9 mm, and annual sunshine hours of 2300–2600 h. The wheat-corn rotation is the main planting pattern in this area.

The experiment adopted a completely randomized block design; the sowing date was 9 October 2020, the planting density was 1.95 million plants/hm^2^, and there were 501 plots in total. Each plot was planted with six rows of new winter wheat varieties, repeated three times, and the plot area was 12 m^2^. The management measures of the experimental field are higher than those of the ordinary field.

### Data collection

#### Global wheat open dataset

The wheat spike image data is a public dataset provided by the Global Wheat Challenge 2021 International Conference on Computer Vision 2021.^1^ The dataset consists of sample_submission.csv, test.zip, and train.zip, which each contain 3,655 images; the resolution of each image is 1024 × 1024.

#### Image data collection

The images were collected at 10:00 a.m. on 19 and 20 April 2021. The weather was clear and cloudless. The smartphone Huawei Honor 20 Pro was used to obtain the wheat heading stage images. The photographer fixed the smartphone on the handheld shooting pole, which shot vertically 50 cm above the wheat canopy. A total of 560 images were taken, and each image has a resolution of 960 × 720. An example of some images at the heading stage of wheat is shown in Figure 1.

#### Dataset construction and labeling

According to the number of images, the wheat heading date image is used as the dataset to construct the wheat spike number YOLOv5s detection model. The training dataset used in this paper is from train.zip provided by global wheat challenge 2021, where train.zip contains 3,655 images of wheat spikes and anchor box files. According to the number of wheat spikes in each image, 500 clear and unobstructed original images of the wheat heading stage were selected as the test set. According to the format requirements of the Pascal VOC dataset, labeling is used to label and generate the dataset XML type annotation file. Cut the original collected image into 640 × 640-pixel images.

#### Data enhancement

In order to improve the generalization ability of the training model, we mainly chose mosaic data enhancement, adaptive anchor box calculation, and adaptive image scaling as data enhancement methods. The details are as follows:

##### Mosaic data enhancement

**FIGURE 2:**
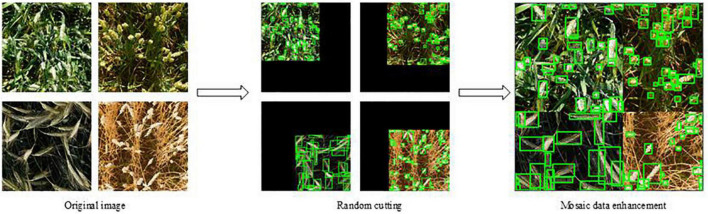
Data enhancement.

Mosaic data enhancement uses four images and stitches them together in the form of random scaling, random clipping, and random arrangement. Each image has its own corresponding annotation box. After stitching the four images, a new image is obtained, and the corresponding annotation box of the image is also obtained. Then the image is transferred to a neural network for learning, which is equivalent to transferring four images for learning, making the model recognize the target in a smaller range. Figure 2 shows the workflow of wheat spikes enhanced with mosaic data.

##### Adaptive anchor box calculation

YOLOv5 network model does not only use the anchor box that has been labeled. Before starting training, it will check the labeled information in the dataset and calculate the best recall rate of the labeled information in this dataset for the default anchor box. When the best recall rate is greater than or equal to 0.98, there is no need to update the anchor box; If the optimal recall rate is less than 0.98, the anchor box that conforms to this data set needs to be recalculated. This function is embedded in the code in YOLOv5. For each training, the best anchor box is adaptively calculated according to the name of the data set. Users can turn off or turn on the image preprocessing function according to their own needs. This paper uses this image preprocessing method before training data.

##### Adaptive image scaling

Due to the different aspect ratios of most images, the size of black edges at both ends is different after using the traditional image scaling method to scale and fill. However, if too much filling is used, there will be a lot of information redundancy, affecting the algorithm’s reasoning speed. In order to further improve the reasoning speed of YOLOv5, this method can adaptively add the fewest black edges to the scaled image.

#### Field measurement data collection

Consistent with the acquisition time of the image data, the measured value of the number of the wheat spikes was collected by an image-based manual counting method. Based on the unified wheat spike counting standard, people with relevant agronomic backgrounds were selected to count, and the average value was taken as the measured value of the wheat spike number corresponding to the image.

### Network model construction

#### YOLOv5s network model

**FIGURE 3:**
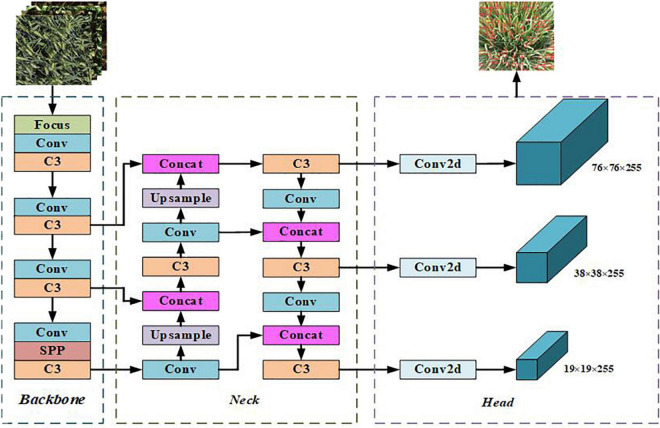
Network structure of YOLOv5s algorithm.

YOLOv5 is the latest product of the YOLO series, which is improved based on YOLOv4, and the running speed has been greatly improved (Chen and Chen, 2022). The YOLOv5 network model structure is mainly divided into four versions: YOLOv5s, YOLOv5m, YOLOv5l, and YOLOv5x. In practical applications, a model of an appropriate size can be selected according to different specific scenarios. YOLOv5 is an improved version based on YOLOv4, which is a one-stage detection network with excellent accuracy and detection speed. After absorbing the advantages of the previous version and other networks, YOLOv5 has changed the previous YOLO target detection algorithm’s characteristics of fast detection speed but low accuracy. YOLOv5 has improved the detection accuracy and real-time performance, meeting the real-time detection needs of video images, and the structure is also more compact. YOLOv5s have the least number of parameters, but the accuracy is low. YOLOv5s have a small depth and width while ensuring high accuracy. The other three versions continue to deepen and widen on this basis, especially when enhancing the extraction of image semantic information. YOLOv5s have the characteristics of fast running speed and high flexibility and have strong advantages in the rapid deployment of models. The network structure is shown in Figure 3. The network consists of four parts: input, backbone, neck, and head. The size of the input image at the input end is 640 × 640 × 3, and the images are preprocessed using strategies such as mosaic data enhancement, adaptive anchor box calculation, and image scaling. The role of the backbone network is to extract rich semantic features from the input image. It includes the Focus module, the Conv module, the C3 module, and the SPP module. In YOLOv5, CSPDarknet53 is used as the backbone network of the model. The neck adopts FPN and PAN to generate feature pyramids, which are used to enhance the detection of multi-scale objects. The head is predicted from the features passed from the neck, and three different scaled feature maps are generated.

#### Improved YOLOv5s network model

Among the five models of the YOLOv5 network, the YOLOv5s model has high accuracy, fewer parameters, and fast detection speed, which can be deployed on hardware devices. The research on wheat spike detection and counting is based on the YOLOV5s network model, and the attention mechanism is added to YOLOV5s to improve the robustness of the network model.

##### Attention mechanism

The introduction of an attention mechanism into convolutional neural networks shows great potential for improving network performance. In the field of computer vision, attention mechanisms are widely used in natural scene segmentation, medical image segmentation, and object detection. Among them, the most representative is the Squeeze-and-Excitation (SE) (Hu et al., 2018), followed by the Convolutional Block Attention Module (CBAM) (Woo et al., 2018) module. Although the SE module can improve the network performance, it will increase the complexity and computational complexity of the model. The CBAM module ignores the channel-space interaction, which leads to the loss of cross-dimensional information. Therefore, this paper selects a more lightweight Efficient Channel Attention (ECA) (Wang et al., 2020) module and a Global Attention Mechanism (GAM) (Liu et al., 2021) that can amplify cross-dimensional interactions. In view of a large number of wheat spikes, dense distribution, occlusion, and overlap in the wheat spike image, the direct use of pre-trained YOLOv5x has high prediction accuracy, but the inference speed of the network is slow, and the number of parameters of the model is 168 M, which is difficult to use in hardware devices Deploy on. The reasoning speed of the YOLOv5s network model is fast, and the number of parameters is small, but the accuracy of YOLOv5s is low. The direct use of the YOLOv5s network model to detect and count wheat spikes is not satisfactory.

##### Introduce the improved C3 module of the efficient channel attention module

**FIGURE 4:**
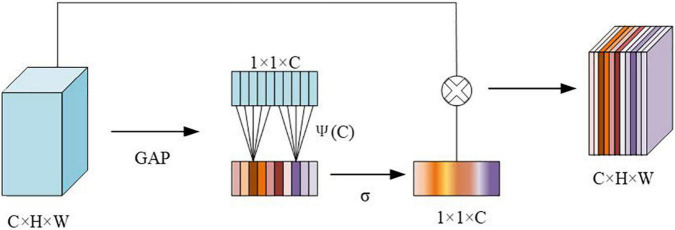
Structure of efficient channel attention (ECA) module.

The ECA module structure is shown in Figure 4. The size of the input feature map is *C* × *H* × *W*, and then the size of the feature map is obtained through Global Average Pooling (GAP). The aggregated features obtained after GAP generate channel weights through a weight-sharing one-dimensional convolution. Among them, the one-dimensional convolution involves the hyperparameter ψ(C), which is the size of the convolution kernel determined by the mapping of the channel dimension C. Then, after the obtained feature map is operated, the output size is 1 × 1 × *C*, and it is multiplied by the corresponding channel of the original input feature, and the final output feature size is *C* × *H* × *W*. Among them, the calculation method is shown in the following formula 1:


(1) $$k=\psi \,\left(C\right)={\left|\frac{\log_{2}\left(C\right)}{\gamma }+\frac{b}{\gamma }\right|}_{o\,d\,d}$$

*C* represents the channel dimension, |*t*|_*odd*_ represents the nearest odd number closest to it *t*, γ is set to 2, and *b* is set to 1.

**FIGURE 5:**
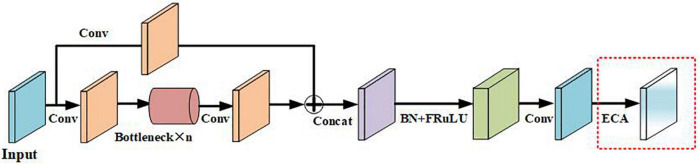
Structure of improved C3 module.

In this study, the ECA module was introduced into the C3 module of the backbone part of the YOLOv5s network model so as to improve useful features, suppress unimportant features, and improve the accuracy of network model detection without additional model parameters. The improved C3 module is named the ECA-C3 module, and its structure is shown in Figure 5.

##### Introduce the YOLOV5s model improved by the global attention mechanism module

**FIGURE 6:**
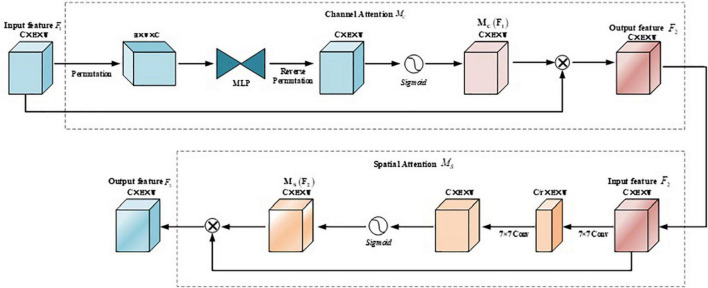
Structure of global attention mechanism (GAM) module.

The purpose of the GAM module is to design an attention mechanism that can reduce information dispersion while amplifying the interactive features of the global dimension. Figure 6 shows the whole process of the GAM module. Given an input feature map, the intermediate states and outputs are defined as follows:


(2) $$F_{2}=M_{c}\,\left(F_{1}\right)⊗F_{1}$$

(3) $$F_{3}=M_{s}\,\left(F_{2}\right)⊗F_{2}$$

Among them, *F*_1_ is the input feature map, *F*_2_ is the feature map obtained after channel attention, *F*_3_ is the final feature map after GAM *M*_*c*_ and *M*_*S*_ represents the channel attention map and the spatial attention map, respectively; ⊗ it represents element-wise multiplication.

The channel attention submodule maintains features in three dimensions using a three-dimensional arrangement and then amplifies the spatial dependencies across dimensions in a two-layer Multi-Layer Perceptron (MLP). In the spatial attention sub-module, first, two convolution operations with a kernel size of 7 × 7 are used for spatial information fusion. At the same time, in order to eliminate the feature loss caused by pooling, the pooling operation is removed here to maintain the feature map further.

### YOLOv5s network model with attention mechanism

**FIGURE 7:**
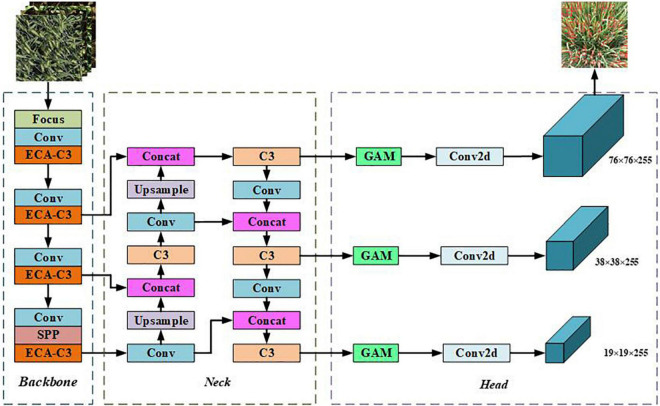
Network structure of improved YOLOv5s algorithm.

The improved YOLOv5s network model is shown in Figure 7. When different from the standard YOLOv5s, the improved model replaces the C3 module of the backbone part with the proposed ECA-C3 module so that the network can effectively extract the target features; GAM is added before the 2D convolution between the neck and head module, and the added GAM will increase the number of parameters of the network model, but it can make the network capture important features like the three-dimensional channel, space width, and space height. The size of the improved YOLOV5s input image is 3 × 640 × 640, and the first prediction branch of the head is used as an example to illustrate. The algorithm structure of the improved YOLOv5s model is shown in Table 1. Among them, “from” refers to the input layer corresponding to the layer module, and −1 refers to the previous layer.

**TABLE 1:** Algorithm structure of improved YOLOv5s.

Number of layers	From	Parameter quantity	Module name
0	−1	3520	Focus
1	−1	18560	Conv
2	−1	18819	ECA-C3
3	−1	73984	Conv
4	−1	115715	ECA-C3
5	−1	295424	Conv
6	−1	625155	ECA-C3
7	−1	1180672	Conv
8	−1	656896	SPP
9	−1	1182723	ECA-C3
10	−1	131584	Conv
11	−1	0	Upsample
12	[−1,6]	0	Concat
13	−1	361984	C3
14	−1	33024	Conv
15	−1	0	Upsample
16	[−1,4]	0	Concat
17	−1	90880	C3
18	−1	147712	Conv
19	[−1,14]	0	Concat
20	−1	296448	C3
21	−1	590336	Conv
22	[−1,10]	0	Concat
23	−1	1182720	C3
24	[17,20,23]	8622262	Detect

#### Channel attention modeling

First, a feature map with a size of 256 × 80 × 80 is obtained through the C3 module, and a feature map of 80 × 80 × 256 is obtained through dimension transformation; the feature map is passed through a two-layer MLP, and the channel scaling rate is set to 4. The dimension of the feature map is reduced to 80 × 80 × 64, and then the dimension is increased to 80 × 80 × 256; the feature map is restored to the original shape and size of 256 × 80 × 80 through dimension transformation; the *sigmoid* function is used to obtain the size of 256 × 80 × 80 channel attention map; multiplies the original input feature map *F* and *M*_*C*_(*F*_1_) to get a feature map of size 256 × 80 × 80.

#### Spatial attention modeling

First, *F*_1_ pass a 7 × 7 convolution, and set the same channel scaling rate as the channel attention, and the size of the obtained feature map is 64 × 80 × 80; then go through a 7 × 7 convolution again to restore the feature map to 256 × 80 × 80. After using the *sigmoid* function, a spatial attention map *M*_*S*_(*F*_2_) with a size of 256 × 80 × 80 is obtained; multiply with *F*_1_ and *M*_*S*_(*F*_2_), an output feature map with a size of 256 × 80 × 80 is obtained.

### Experimental results and analysis

#### Experimental equipment and parameter settings

The experiment is based on the deep learning framework built by Pytorch1.10 and CUDA11.2, using Linux Ubuntu18.04 LTS operating system, Intel^®^ Core ™i7-8700 CPU @3.70GHZ processor, Tesla T4 16G for experiments. The size of the images for training, verification, and testing in this experiment is 640 × 640 pixels, the input batch size is set to 8, and the training process is set to 60 epochs. The training process uses the stochastic gradient descent (SGD) optimizer; the initial learning rate is 0.01, the momentum factor is 0.937, and the weight decay rate is 0.0005.

#### Evaluation index and loss function

YOLOv5s, YOLOv5m, YOLOv5l, YOLOv5x, and improved YOLOv5s are validated on the validation set randomly divided into the public data set Global wheat challenge 2021, and the evaluation indicators Precision, Recall, mAP@0.5, and mAP@0.5:0.95 are similar, it showed that all three models could achieve the best performance in the detection task of the Global wheat challenge 2021, so the above four evaluation indicators are not selected to evaluate the model. This study mainly evaluates the performance of the model when the wheat spike data collected in the field is used as the test set for wheat spike counting. Therefore, the accuracy (Accuracy, ACC) is selected as the evaluation index for YOLOv5s counting, using the number of parameters and the amount of calculation (GFLOPs) and inference speed to evaluate model performance. The calculation formula of accuracy is as follows:


(4) $$A\,C\,C=\frac{T\,P+T\,N}{T\,P+F\,N+F\,P+T\,N}$$

(5) $$R\,e\,c\,a\,l\,l=\frac{T\,P}{T\,P+F\,N}$$

(6) $$\mathrm{mAp}=\int_{0}^{1}P\cdot R\,dR$$

Among them, *TP* they represent true positives, *TN* represents true negatives, *FP* represents false positives, and *FN* represents false negatives. The larger the ACC value, the better the detection effect of the model.

In this study, CIoU is selected as the loss function to calculate the localization loss. CIoU can better represent the gap between the prediction and annotation frames, making the network model more robust during training. The CIoU loss function is defined as follows:


(7) $$\text{IoU}=\frac{a\,r\,e\,a\,\left(a\,r\cap t\,r\right)}{a\,r\,e\,a\,\left(a\,r\cup t\,r\right)}$$

(8) $$\text{CIoU}=1-\text{IoU}+\frac{\rho^{2}\,\left(b,b^{g\,t}\right)}{c^{2}}+\alpha \,v$$

(9) $$\alpha =\frac{v}{\left(1-\text{IoU}\right)+v}$$

(10) $$v=\frac{4}{\pi^{2}}\,{\left(\arctan\,\frac{w_{\mathrm{gt}}}{h_{\mathrm{gt}}}-\arctan\,\frac{w}{h}\right)}^{2}$$

Among them, ar and tr represent the anchor box and the bounding box ρ^2^(*b*,*b^gt^*) and the Euclidean distance between the center points of the anchor box and the bounding box, respectively. α is an equilibrium parameter and does not participate in gradient calculation; ν is a parameter used to measure the consistency of aspect ratio. ^W_gt_^ and ^h_gt_^ are the width and the height of the bounding box, while w and h are the widths and the height of the anchor box.


(11) $$R\,M\,S\,E=\sqrt{\frac{1}{N}\,\sum_{i=1}^{N}{\left(p_{i}-q_{i}\right)}^{2}}$$

(12) $$M\,A\,E=\frac{1}{N}\,\sum_{i=1}^{N}\left|p_{i}-q_{i}\right|$$

where *N* is the number of images, *p*_*i*_ is the angle of the oriented detection box in the *i* image, and *q*_*i*_ is the angle of the corresponding oriented bounding box.

### Quantitative analysis of experimental results

YOLOv5s, YOLOv5m, YOLOv5I, YOLOv5x, improved YOLOv5s, and the Faster R-CNN were used to evaluate the performance metrics of wheat spike data collected in the field. It can be seen from Table 2 that the evaluation metrics of Faster R-CNN were the worst. The evaluation metrics of improved YOLOv5s were better than those of standard YOLOv5s, YOLOv5m, and YOLOv5I and were similar to those of YOLOv5x.

**TABLE 2:** Test performance comparison of different models.

Methods	RMSE	MAE	Recall	mAP@.0.5	Map@.0.5:0.95
YOLOv5s	53.23	41.24	0.887	0.949	0.526
YOLOv5m	51.56	40.83	0.894	0.949	0.522
YOLOv5l	49.71	38.87	0.888	0.947	0.525
YOLOv5x	44.51	33.62	0.913	0.950	0.541
Improved YOLOv5s	43.94	34.36	0.911	0.951	0.545
Faster R-CNN	94.57	87.10	0.819	0.862	0.355

The evaluation metrics of the average error rate and AP rate of the above different models on the test images are shown in Table 3. YOLOv5x has the highest AP, and Faster R-CNN has the lowest AP. Compared with the standard YOLOv5s, the accuracy of the improved YOLOv5s is improved by 4.95%, and compared with YOLOv5m and YOLOv5l, the AP is improved by 4.32 and 2.50%, respectively, and the AP is basically close to that of YOLOv5x.

**TABLE 3:** Statistical average error and average accuracy.

Methods	Mean error (%)	Mean accuracy (%)
YOLOv5s	33.34%	66.66%
YOLOv5m	33.29%	67.29%
YOLOv5l	30.89%	69.11%
YOLOv5x	27.52%	72.48%
Improved YOLOv5s	28.39%	71.61%
Faster R-CNN	54.07%	45.93%

Table 4 shows the comparison of different models in parameter quantity, giga floating-point operations per second (GFLOPs), inference, inference speed, and graphic processing unit (GPU) resource occupancy. Although the standard YOLOv5s parameter quantity, GFLOPs, inference, inference speed, and GPU resource occupancy are the least, the detection accuracy is low. While Faster R-CNN has the most GFLOPs, inference, inference speed, and GPU resource occupancy, the effect is the worst. The parameter quantity, GFLOPs, inference, inference speed, and GPU resource occupancy of the improved YOLOv5s are all larger than those of the standard YOLOv5s and less than those of the standard YOLOv5I and YOLOv5x.

**TABLE 4:** Comparison of parameter quantity, GFLOPs, inference, inference speed, and GPU resource occupancy of different models.

Methods	Parameter quantity (M)	GFLOPs	Inference (Min)	Inference speed (ms)	GPU resource occupancy (G)
YOLOv5s	13.38	15.8	370.5	7.5	1.70
YOLOv5m	39.77	47.9	396.2	11.6	1.80
YOLOv5l	87.90	107.6	415.6	17.3	2.10
YOLOv5x	164.36	204.0	479.9	29.0	2.40
Improved YOLOv5s	28.81	31.6	372.5	14.7	2.42
Faster R-CNN	41.30	278.2	755.3	227.7	7.87

Table 5 compares the AP and training time between EloU and CloU. By comparing the effects of EloU and CloU in the YOLOv5s model, the AP after using EloU is slightly higher than that of CloU, but the training time is significantly increased. Therefore, this paper selects CloU as the loss function to calculate the localization loss.

**TABLE 5:** Comparison of average accuracy and training time between CloU and EloU of YOLOv5 models.

Methods	Mean accuracy (%)	Inference (Min)
Improved YOLOv5s with CIoU	71.61%	372.5
Improved YOLOv5s with EIoU	72.82%	405.6

### Qualitative analysis of experimental results

**FIGURE 8:**
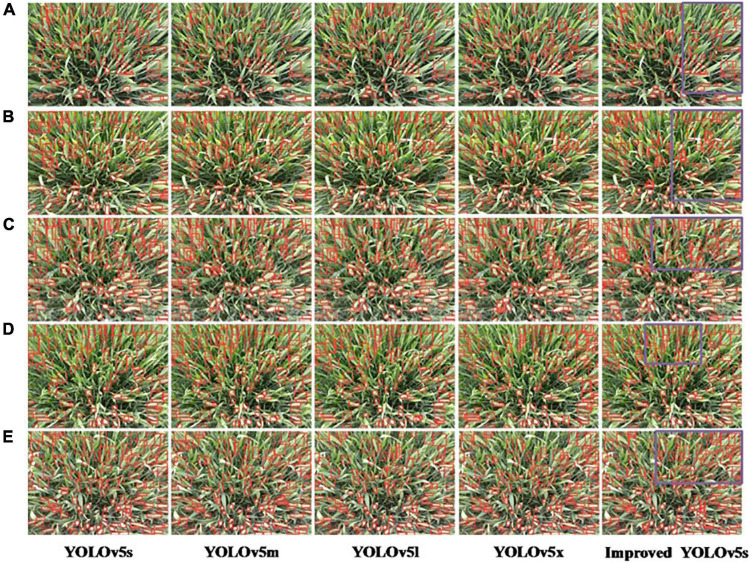
Qualitative analysis of experimental results of YOLOv5 algorithm. **(A–E)** Represent the number of images.

Figure 8 compares the recognition results of the standard YOLOv5s and YOLOv5m network models with the improved YOLOv5s network model in this paper for the recognition of wheat spikes in the field environment. It can be seen from Figure 8 that the standard YOLOv5s, YOLOv5m, YOLOv5l, and YOLOv5x network models have seriously missed detections in areas with dense wheat spikes. With a high recognition rate and good generalization performance, the purple box area shows the superiority of the improved YOLOv5s detection results.

**FIGURE 9:**
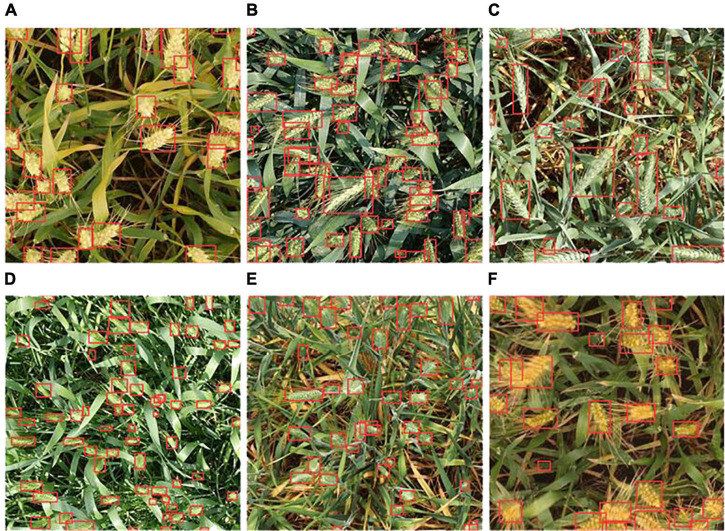
Experimental effects of improved YOLOv5s under different densities and backgrounds. **(A–F)** Represent six different images randomly selected from the global wheat challenge 2021 International Conference on computer vision 2021 dataset.

The images of wheat spikes are dense and sparse. Figure 9 shows the experimental results of the improved YOLOv5s model under different densities and backgrounds. Figures 9A,F show the counting results when the spikes of wheat are sparse; Figures 9B–D show the counting results in the case of dense wheat spikes. Among them, the color of wheat leaves in Figures 9B,D is similar to that of wheat spikes, and the color of wheat leaves in Figures 9C,E is yellow, and the color of wheat spikes is green.

## Discussion

Spike number is an important indicator for determining wheat yield phenotypic traits, and spike detection is a hot spot in wheat phenotype research (Fernandez-Gallego et al., 2019). The wheat spike image data comes from the heading stage of this study. At this time, due to the large difference in the shape and the high density of the wheat spike, there are too many occluded parts, and the characteristics of the wheat spike are not obvious. In the process of spike recognition, there is a problem of omission in the detection of wheat spike occlusion, which leads to an error in the wheat spike count. In the wheat spike detection, the overlapping wheat spike in some images is not identified and marked, the adjacent wheat spike is not identified and marked, and the two wheat spikes are closely connected and identified as one wheat spike. This study proposes a target detection based on improved YOLOv5s, which corrects these problems in the process of wheat spike recognition. It effectively solves the problem of missed detection caused by occlusion and overlap in wheat spike detection. Therefore, the target detection method based on the improved YOLOv5s significantly improves the accuracy and recognition ability of the wheat spike in the image.

Deep learning is currently the main technical means of wheat spike recognition, detection, and counting. Using digital images of winter wheat to obtain the color, texture, and shape features of a wheat spike and establishing a wheat spike recognition classifier through deep learning methods, we identified wheat spike recognition and detection and counting. Zhou et al. (2018b) proposed an SVM segmentation method for segmenting wheat spikes in visible light images. Sadeghi-Tehran et al. (2019) developed the wheat spike number counting system DeepCount, which is used to automatically identify and count the number of wheat spikes in the images of wheat spikes. Alkhudaydi et al. (2019b) and Misra et al. (2020) constructed the SpikeletFCN spikelet counting model based on a fully convolutional network, which used the density estimation method to calculate the number of wheat spikelets. These research results show that the deep convolutional neural network has good robustness for wheat spike counting. In this study, when the resolution of the input image is higher, the detection accuracy is also higher, which is consistent with other research results tested on general datasets (Singh et al., 2018). This study introduces ECA in the C3 module of the backbone structure of the YOLOv5s network model. The GAM module is inserted between the neck structure and the head structure. The accuracy and efficiency of the improved YOLOv5s target detection method are significantly improved, which solves the problem of wheat spikes caused by cross occlusion to a certain extent. The problem of unclear and omitted spike identification has better practical application value.

## Conclusion

We developed an improved YOLOv5s-based attention mechanism for wheat spike number image detection. The method includes three key steps: data preprocessing of the wheat spike image, adding an attention mechanism module for network improvement, and YOLOv5s network model fused with an attention mechanism. In the wheat spike counting task, the accuracy of the improved YOLOv5s model reached 71.61%, which was 4.95% higher than that of the standard YOLOv5s model and had higher counting accuracy. The improved YOLOv5s and YOLOv5m have similar parameters, while RMSE and MEA are reduced by 7.62 and 6.47, respectively, and the performance is better than YOLOv5l. The experimental results show that the improved YOLOv5s algorithm improves the applicability in complex field environments, which can accurately detect the number of small-scale wheat spikes and better solve the occlusion and overlapping problems of a wheat spike.

In the case of extremely dense samples, the coincidence probability of wheat spike heads is high, and the regression idea of the YOLO algorithm is based on dividing the image into grids; that is, each grid can only predict one target at most, so it does not perform well when there are multiple target objects in the same grid, and it is impossible to identify all the targets. Due to its portability and lightweight network, YOLOv5s is used as the main model for training, which improves its flexibility and speed compared with YOLOv4, and reduces many of its parameters to make it applicable to portable devices. The improved model needs to take into account the training accuracy and training speed and increase the number of parameters.

The improved YOLOv5s method proposed in this study can realize the counting of wheat spikes and can meet the needs of high-throughput operations in the wheat field environment. In future research work, we will gradually optimize the built-in YOLOv5s network structure and analyze the wheat spike detection network structure for the wheat spike images acquired by smartphones to obtain better wheat detection performance. In addition, we will envisage applying this method to other crop counts to demonstrate its robustness in solving occlusion and overlap problems. Subsequently, the improved YOLOv5s method can save time and effort.

## Data availability statement

The raw data supporting the conclusions of this article will be made available by the authors, without undue reservation.

## Author contributions

HZ and LR: conceptualization, software, formal analysis, and visualization. HZ: methodology, writing – original draft preparation, and supervision. HZ, MZ, DC, and QZ: validation. HZ and YW: investigation. HZ, GZ, and GL: resources. HZ, YW, and LR: data curation. YW and GL: writing – review and editing. HZ and GZ: project administration and funding acquisition. All authors have read and agreed to the published version of the manuscript.

## Footnotes

1 https://www.aicrowd.com/challenges/global-wheat-challenge-2021/dataset_files, 6 July 2021, day download.
